# Role of Contrast-Enhanced Ultrasound in the Follow-Up after Endovascular Abdominal Aortic Aneurysm Repair

**DOI:** 10.3390/diagnostics12123173

**Published:** 2022-12-15

**Authors:** Filippo Benedetto, Domenico Spinelli, Francesco La Corte, Narayana Pipitò, Gabriele Passari, Giovanni De Caridi

**Affiliations:** Dipartimento di Scienze Biomediche, Odontoiatriche e delle Immagini Morfologiche e Funzion-ali, Università di Messina, 98122 Messina, Italy

**Keywords:** contrast-enhanced ultrasound, duplex ultrasound, abdominal aortic aneurysm repair, endoleak, follow-up

## Abstract

Background: The aim of this study was to assess whether contrast-enhanced ultrasound (CEUS) shows a false negative rate close to zero and therefore is suitable as the main non-invasive follow-up strategy for long-term monitoring after endovascular aortic repair (EVAR). Methods: We included all consecutive patients who underwent CEUS as follow-up after EVAR at our center between January 2017 and December 2021.The follow-up protocol consisted of Duplex ultrasound (DUS) with CEUS at 1, 3, 6 months postoperatively and every 6 months thereafter. Results: A total of 125 patients underwent 228 CEUS. The aneurysm sac showed shrinkage in 80 (64%) patients, stability in 32 (25.6%), and enlargement in 13 (10.4%). A total of 29 (23.2%) patients showed type 2 endoleak, 6 (4.8%) patients showed type 1 endoleak and 3 (2.4%) patients showed type 3 endoleak. Thirteen patients underwent one or more reinterventions. The sensitivity of CEUS vs. DUS was 100% vs. 75% (*p* > 0.0001). In classifying type 2 endoleak, CEUS compared to DUS showed a sensitivity of 93.2% vs. 59.4% and a specificity of 99.3% vs. 99.3%. CEUS showed a higher sensitivity compared to DUS in the detection of type 2 endoleak. CEUS permits the identification of a subset of patients requiring a stricter follow-up protocol.

## 1. Introduction

Endovascular abdominal aortic aneurysm repair (EVAR) has several advantages over traditional open surgical aneurysm repair, including lower invasivity and shorter hospital stay [1]. However, its main drawback is the need for a life-long follow-up [2]. Possible complications include endograft migration, kinking, fracture, thrombosis, and endoleak, which is aneurysm sac reperfusion. This can be due to loss of the proximal or distal sealing (type Ia and Ib endoleak, respectively), to retrograde perfusion through side branches (type II endoleak), to endograft component disconnection or rupture (type III endoleak). Clinical practice protocols regarding the optimal follow-up strategy are heterogeneous, as there is no agreement as to which method should be preferred [3,4,5]. In fact, computed tomography angiography (CTA) has good accuracy and reproducibility, but it is burdened by high cumulative dose radiation exposure and contrast agent nephrotoxicity, especially if used annually over a long period of time. The average radiation dose absorbed during a 5-year post-EVAR follow-up protocol based on CTA is not negligible, being estimated at approximately 145–205 mSv [6]. Compared to patients undergoing open aneurysm repair, patients undergoing EVAR have been shown to present a faster decline in renal function during the follow-up, which is likely to be attributable to the higher number of CTA required for the long-term surveillance [7]. On the contrary, duplex ultrasound (DUS) has virtually no side effects, but its reliability and reproducibility are lower [8]. On the other hand, contrast-enhanced ultrasound (CEUS) has appealing advantages over DUS, showing better performance in detecting endoleak, with a very low risk for the patient, mainly related to allergic reactions, and a significantly lower cost compared to CTA [9,10]. In fact, the sustainability of a post-EVAR surveillance protocol based on CTA, in terms of cost-effectiveness, has been questioned [10]. Moreover, being a dynamic test, CEUS might show an even better sensitivity than CTA in detecting and correctly classifying endoleaks [11,12]. In particular, CEUS may outperform CTA in the assessment of hypo- dynamic or low-flow endoleaks [12]. Additionally, from an organizational standpoint, CEUS presents less criticality than CTA, especially in patients with chronic kidney disease [3]. The aim of this study is to assess whether CEUS shows a false negative rate close to zero and therefore is suitable as the main non-invasive follow-up strategy for long-term monitoring after EVAR.

## 2. Materials and Methods

### 2.1. Study Design and Setting

A retrospective cohort study was conducted including all consecutive patients who underwent at least one CEUS exam as follow-up after EVAR at the Unit of Vascular Surgery of Gaetano Martino Teaching Hospital, University of Messina between 2017 and 2021. Exclusion criteria were ruptured aneurysms and isolated iliac artery aneurysm. Primary endpoint was the comparison between CEUS and DUS. Secondary endpoint was the detection of endoleak type 2 with slow inflow (T2EL). This study was approved by the Ethical Review Board of our institution, and written informed consent was obtained from all patients. 

### 2.2. Data Collection

Data collected at baseline included sex, age, ASA classification, and aortic endografts with suprarenal or infrarenal fixation.

### 2.3. Variables

Collected variables were demographic data, comorbidities, maximum aneurysm diameter preoperatively, operative details, date of follow-up control, aortic diameter at follow-up, presence of aneurysmal sac shrinkage, presence of endoleak, other complications, reintervention or death. The detection and identification of endoleak type were recorded for each imaging method used (DUS, CEUS, CTA, and angiography).

### 2.4. Follow-Up Protocol

The follow-up protocol consisted of DUS with CEUS at 1, 3, 6 months postoperatively and every 6 months thereafter. In the case of consistent aneurysm sac shrinkage over time, when the operator considered CEUS not necessary, only DUS was performed. All patients underwent CTA at 1 and 12 months and when deemed necessary or indicated by the operator.

**Figure 1:**
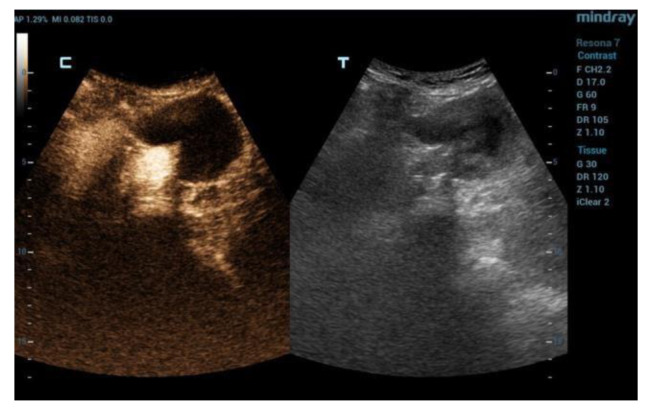
Initial control phase of EVAR with CEUS (c) and DUS (t). Normal perfusion endoprosthesis and no T2EL detected (c).

**Figure 2:**
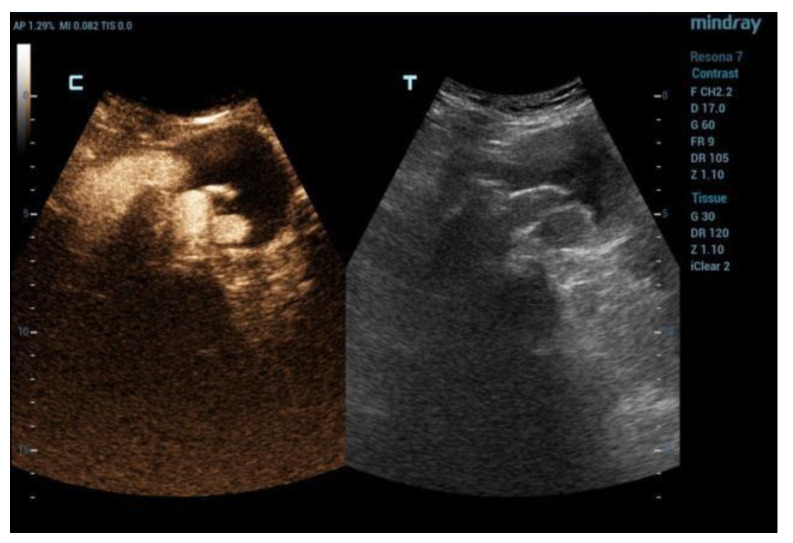
Tardive control phase of EVAR with CEUS (c) and DUS (t). Evidence of T2EL with low washout (c).

**Figure 3:**
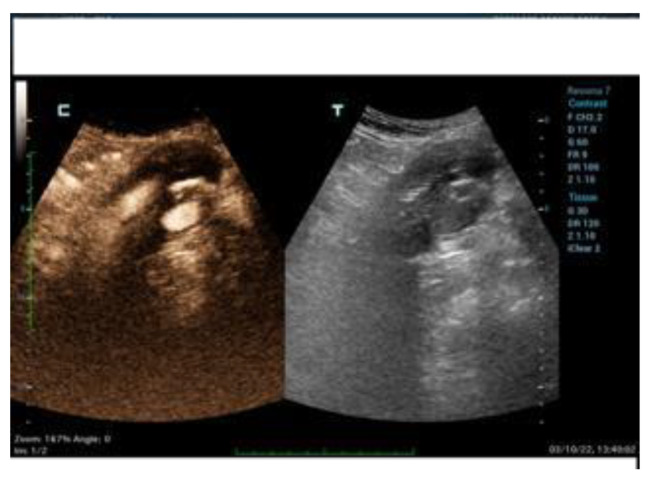
Evidence of T2EL with CEUS (c) and DUS (t).

**Figure 4:**
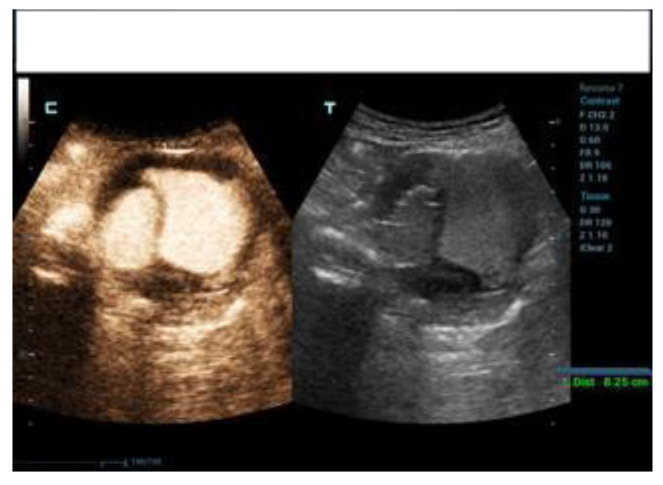
Evidence of T1EL type A with CEUS (c) and DUS (t).

**Figure 5:**
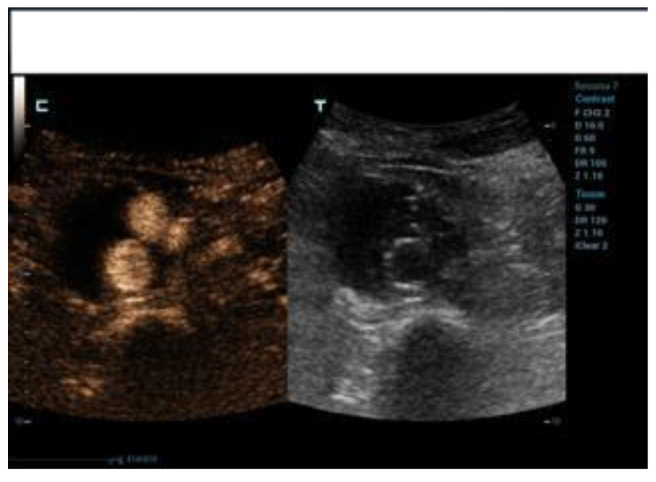
Evidence of T1EL type B with CEUS (c) and DUS (t).

The DUS and CEUS examinations were all performed using a Resona 7 Ultrasound System (Mindray Bio- Medical Electronics Co., Ltd., Shenzhen, China), using a 1–5 MHz multifrequency curved-array transducer (SC-5-1U, Mindray Bio-Medical Electronics Co., Ltd., Shenzhen, China). The patients were advised to avoid taking anything by mouth on the day of the exam and to avoid fiber intake in the previous three day sand to take simethicone oral drops in order to limit bowel gas. The exam started with the DUS in B-mode scanning the aneurysm sac both in transversal and sagittal imaging. This allowed the operator to measure the aneurysm sac diameter and to search for any anomalies of the endograft (e.g., interruption of endograft wall continuity). Subsequently, color-coded DUS was used to search for signals suggestive of endoleak inside the aneurysm sac, and to assess the regular flow inside the endograft main body and branches, which was confirmed by spectral doppler waveform analysis. Spectral doppler was used to investigate the flow through a patent inferior mesenteric artery and any flow in the aneurysm sac suggesting endoleak. Then, after switching to CEUS specialized software, with a dual-screen format (B-mode display on the left and contrast display on the right to guide anatomic landmarks), 2.5 cc of 2nd-generation contrast medium consisting microbubbles of sulfur hexafluoride stabilized by a phospholipid shell (SonoVue; Bracco, Milan, Italy) was administered intravenously and a 3-min clip was acquired, again scanning the aneurysm sac in transversal and sagittal view, searching for signs of endoleak (i.e., contrast-enhancement in the residual sac outside the endograft). A low mechanical index was used (0.2), and the focal zone was placed just beyond the aorta in order to minimize bubble destruction. An endoleak was diagnosed when contrast enhancement of the aneurysm sac outside of the endograft was detected. A synchronous enhancement was suggestive of type 1 (T1EL) or 3 (T3EL) endoleak, while a delayed enhancement was suggestive of type 2 (T2EL) endoleak. The characteristics of this finding have been classified in the presence of EL and type I, II, III, and IV. In the case of EL II, the timing of appearance (inflow = time from injection of the contrast medium to appearance in the bag) and synchrony with the vascularization of the graft was evaluated: rapid within 60 s; late after 60 s. The timing of outflow was also evaluated (from the injection of the contrast medium to its disappearance from the bag): rapid within 3 min and late after 3 min. EL type II with inflow <60 s and rapid outflow (<3 min) were defined as “hyperdynamic”. Those with slow inflow (>60 s) and slow (>3 min) or no washout were defined as “hypodynamic” (Figure 1, Figure 2 and Figure 3). In order to clarify the origin and the direction of the endoleak, the proximal and distal sealing zones were examined, as well as the lumbar arteries (LA), the sacral artery (SA) and the inferior mesenteric artery (AMI). When the examination was suggestive of a type 1 (Figure 4 and Figure 5) or 3 endoleak or when sac enlargement ≥ 5 mm was detected, a CTA was carried out.

### 2.5. Statistical Analysis

Continuous variables were reported as the mean (±standard deviation) or as the median (interquartile range, IQR) as appropriate. Categorical variables were reported as counts (percentage). The sensitivity, the specificity, the positive predictive value (PPV) and the negative predictive value (NPV) were calculated using true positive, true negative, false positive and false negative rates calculated for each CEUS exam using the closest CTA available as the reference standard. In the case of doubt requiring angiography, this was used as reference.

## 3. Results

We reviewed the data of consecutive patients undergoing CEUS for EVAR follow- up at our outpatient clinic between January 2017 and December 2021. A total of 125 patients underwent CEUS at least once during their follow-up after EVAR and were included in this study. The majority of patients were male (*n* = 114, 91%) and the mean age was 74.6 ± 7.3. These patients underwent, in total, 228 CEUS exams during the study period. No contrast medium-related adverse events were observed.

### 3.1. Outcome Data

During a median follow-up of 19 months (IQR 8–35 months), 80 (64%) patients showed shrinkage, 32 (25.6%) patients showed aneurysm sac stability, and 13 (10.4%) showed sac enlargement. The mean preoperative aneurysm sac diameter was 56 ± 13 mm. The mean aneurysm diameter at last follow-up visit was 50 ± 15 mm. A total of 29 (23.2%) patients showed type 2 endoleak (24 with rapid washout and 5 with slow washout), 6 (4.8%) patients showed type 1 endoleak and patents 3 (2.4%) patients showed type 3 endoleak; 13 (10.4%) patients underwent reintervention, with 4 undergoing reintervention multiple times. The indications for the reinterventions were the following: T1EL in eight cases (four 1A, three 1B, one 1C), T3EL in six cases, and T2EL with sac enlargement in five cases (in all cases with slow washout) (Table 1).

### 3.2. Main Results

In detecting any type of endoleak, the sensitivity of CEUS vs. DUS was 100% vs. 75% (McNemar chi-square test, *p* > 0.0001). The classification of endoleak type was uncertain in five cases of type 2 endoleak and in one case of type 3 endoleak. The diagnosis was clarified by analyzing the CEUS and CTA images and discussing the cases in a multidisciplinary team, or was confirmed by angiography. As far as type 2 endoleak identification is concerned, CEUS compared to DUS showed a sensitivity of 93.2% vs. 59.4%, a specificity of 99.3% vs. 99.3%, a PPV of 98.6% vs. 97.7%, and a NPV of 96.8% vs. 83.6%. In the detection of type 1 or 3, CEUS and DUS did not show any discrepancies. Both techniques had a sensitivity of 84,6%, a specificity of 100%, a PPV of 100% and a NPV of 99.1% (Table 2).

## 4. Discussion

After EVAR, lifelong surveillance is necessary, as complications involving the risk of rupture may occur at any time during the follow-up [13,14]. Mulay et al. showed no difference in overall survival between patients who underwent EVAR with or without T2EL and the patients who underwent a secondary intervention did not have better survival compared with those who did not undergo a secondary intervention [15]. CTA is considered the mainstay of follow-up imaging after EVAR [3]. However, the routinary use of repeated CTA exams in EVAR follow-up is debated, as this exposes the patient to a high cumulative dose of radiation and increased risk of renal function impairment [16]. Furthermore, CTA does not provide dynamic flow information; in fact, only DUS is able to document velocity and direction flow in the aneurysm sac and branch vessels entering the aneurysm. Our study showed that CEUS improved the performance of ultrasound, particularly for the detection of type 2 endoleak. Type 2 endoleak is associated with increased risk of sac expansion, late intervention and aneurysm-related death [14]. Approximately 50% of patients with late or persistent type 2 endoleak experience sac expansion [17]. For this reason, the detection of type 2 endoleak may be crucial for the identification of a subset of patients requiring a stricter follow-up protocol [3]. Some authors found that a certain amount of endoleaks did not become evident until two or more years had passed. The reasons for the late appearance of a type 2 endoleak remain unclear. Although some of these patients show type 2 endoleak after an initial shrinkage, others present with endoleak after a period of sac diameter stability [14]. In such cases, a misdiagnosed early low-flow endoleak cannot be excluded. We can speculate that in such cases, high-sensitivity exam ination, such as CEUS, could improve the detection rate of a low-flow type 2 endoleak. This could explain the relatively high rate of early endoleaks observed in our study. Another possible explanation for late-onset endoleak is the presence of an intermittent position-dependent endoleak. This entity, which has been reported by some authors [18,19], can be potentially detected, when suspected, with CEUS, whereas CTA cannot detect it. Indeed, although CTA is used as a reference exam in many studies, CEUS may well show a sensitivity even higher than CTA in low-flow endoleaks [8]. In fact, artifacts associated with the metal stent struts and radiopaque markers may hide a small endo-leak [20]. Indeed, some authors pointed out that beam-hardening artifacts may hide small-sized endoleaks [21]. In this setting, CEUS may help clarify a finding of dubious interpretation at CTA. In addition, CEUS may help identify the inflow and outflow vessels of a type 2 endoleak, which may be relevant for the planning of the treatment [22]. Some authors proposed post-EVAR surveillance protocols based on CTA and DUS, with CEUS (or 3D CEUS) only used when an endoleak was detected [18]. Others are in favor of a switch of the preferred examination from CTA to CEUS [13]. Similarly, we preferred to opt for a more extensive use of CEUS. In our protocol, every patient under- goes at least one CEUS examination, and CEUS is the technique of choice for the follow- up of patients with type 2 endoleak or gutter endoleak. This approach allows multiple operators to perform a sufficient number of CEUS examinations each, and therefore to acquire experience and confidence with this technique. The extensive use of CEUS for the follow-up after EVAR appears justified in light of recent evidence showing a high sensitivity and a similar specificity of CEUS even when compared to CTA [11]. Moreover, some authors have pointed out that, compared to endoleaks missed by CEUS, endoleaks missed by DUS are more likely to have clinical relevance [23]. Similarly, in our experience, in two of the patients with persistent type 2 endoleak, which later led to a sac diameter increase and indication for reintervention, the endoleak was missed by DUS and detected by CEUS. This information is relevant, as patients with persistent type 2 endoleak undergo a stricter follow-up schedule than patients with no endoleak [3]. Mazzacaro et al. reported a higher percentage of sensitivity and specificity of DUS to detect endoleaks than our series but with the difference that the number of T1ELs reported is significantly higher than in our experience [24]. CEUS permits high-sensitivity detection of the presence of hyperdynamic or hypodynamic type II endoleaks and endoleak with a slow washout can be considered as a predictive factor of the increase in volume and sac diameter and then for reintervention.

### Limitations

There are some limitations in this study. Considering that the number of positive cases (i.e., patients with endoleak) is limited, further studies with a larger number of patients would be desirable in order to confer more robustness to the results. The interpretation of CEUS, as for other ultrasound-based imaging techniques, may differ among operators and the quality of the examination may vary with the experience of the operator. In our study, the majority of the examinations were carried out by one experienced operator (DB), but some of the examinations were carried out by operators with less extensive experience. For this reason, some degree of heterogeneity among the examination quality cannot be excluded. However, each operator was supported by an experienced operator during the early phase of his learning curve. Since this is a retrospective study, there is an intrinsic risk of bias. Moreover, there was no blinding in the interpretation of CEUS with regard to DUS. In some cases, the CTexam was not synchronous with the corresponding CEUS exam, being up to 30 days apart. As endoleaks may change over time, there is a theoretical risk of misinterpreting such occurrences as false positives or false negatives.

CEUS showed a higher sensitivity compared to DUS in the detection of type 2 endoleak. For this reason, it is a valuable tool in the follow-up of patients undergoing EVAR, as it permits the identification of a subset of patients requiring a stricter follow-up protocol.

## Figures and Tables

**Table 1:** Reinterventions in patients with endoleak.

	First Intervention	Endoleak Type	CEUS	DUS	Early vs. Late Endoleak	Reintervention
1	EVAR + bilateral IBD	1C	+	+	late	Implantation of covered BES landing in hypogastric artery
		3 *	+	+	late	Implantation of bridging endograft
2	EVAR + right IBD and left bell bottom	2 *	+	-	late	Coil embolization of sacral artery
3	EVAR with rightdouble barrel	3	+	+	early	Ballooning of overlap areas
4	Chimney-EVAR	1A	+	+	early	Proximal extension
5	EVAR + right IBD and left double barrel	3	+	+	early	Relining with extension in left external iliac artery
6	EVAR	2	+	+	late	Coil embolization
7	EVAR	1B	+	+	late	Distal extension
8	EVAR	2	+	+	late	Coil embolization
	EVAR	1B	+	+	late	Distal extension
9	EVAR + left IBD	2 *	+	+	late	Coil embolization
		2	+	+	unresolved	Laparoscopic clipping
		2	+	+	unresolved	Open conversion
10	EVAR + left double barrel + right hypogas tric embolization	3	+	+	late	Relining of left double barrel
		3	+	+	unresolved	Relining with exclusion of left hypogastric artery
		3 + 1A			unresolved	Proximal extension, relining
		1A			early	Open conversion
11	EVAR	2	+	-	early (persistent)	Coil embolization
12	Aorto-uni-iliac	1A	+	+	early	Proximal extension with chimney
13	EVAR	1B	+	+	late	Distal extension with exclusion of left hypogastric artery

CEUS, contrast-enhanced ultrasound; DUS, duplex ultrasound; EVAR, endovascular abdominal aortic aneurysm repair; IBD, iliac branch device; BES, balloon-expandable stent; * classification uncertain, confirmed with angiography; + CEUS or DUS positive for endoleak, - CEUS or DUS negative for endoleak.

**Table 2:** Endoleaks identified by contrast-enhanced ultrasound (CEUS) examination during the follow-up period; the sensitivity, the specificity, the positive predictive value and the negative predictive of duplex ultrasound (DUS) vs. CEUS. EL = endoleak.

		Endoleak Type			
		Type Ia	Type Ib	Type II	Type III
		2	4	29	3
		Sensitivity (%)	Specificity (%)	Positive predictive value (%)	Negative predictive value (%)
DUS	Any type EL	75	-	-	-
	Type I and III EL	84.6	100	100	99.1
	Type II EL	93.2	99.3	98.6	96.8
CEUS	Any type EL	100	-	-	-
	Type I and III EL	84.6	100	100	99.1
	Type II EL	59.4	99.3	97.7	83.6

## Data Availability

The data that support the findings of this study are available from the corresponding author, [F.B. and G.D.C.], upon reasonable request.
